# Is expanding HPV vaccination programs to include school-aged boys likely to be value-for-money: a cost-utility analysis in a country with an existing school-girl program

**DOI:** 10.1186/1471-2334-14-351

**Published:** 2014-06-26

**Authors:** Amber L Pearson, Giorgi Kvizhinadze, Nick Wilson, Megan Smith, Karen Canfell, Tony Blakely

**Affiliations:** 1 Department of Public Health, University of Otago, 23A Mein Street, Wellington 6242, New Zealand; 2 Cancer Modelling Group, Prince of Wales Clinical School, University of New South Wales, Sydney, Australia

## Abstract

**Background:**

Similar to many developed countries, vaccination against human papillomavirus (HPV) is provided only to girls in New Zealand and coverage is relatively low (47% in school-aged girls for dose 3). Some jurisdictions have already extended HPV vaccination to school-aged boys. Thus, exploration of the cost-utility of adding boys’ vaccination is relevant. We modeled the incremental health gain and costs for extending the current girls-only program to boys, intensifying the current girls-only program to achieve 73% coverage, and extension of the intensive program to boys.

**Methods:**

A Markov macro-simulation model, which accounted for herd immunity, was developed for an annual cohort of 12-year-olds in 2011 and included the future health states of: cervical cancer, pre-cancer (CIN I to III), genital warts, and three other HPV-related cancers. In each state, health sector costs, including additional health costs from extra life, and quality-adjusted life-years (QALYs) were accumulated. The model included New Zealand data on cancer incidence and survival, and other cause mortality (all by sex, age, ethnicity and deprivation).

**Results:**

At an assumed local willingness-to-pay threshold of US$29,600, vaccination of 12-year-old boys to achieve the current coverage for girls would not be cost-effective, at US$61,400/QALY gained (95% UI $29,700 to $112,000; OECD purchasing power parities) compared to the current girls-only program, with an assumed vaccine cost of US$59 (NZ$113). This was dominated though by the intensified girls-only program; US$17,400/QALY gained (95% UI: dominant to $46,100). Adding boys to this intensified program was also not cost-effective; US$128,000/QALY gained, 95% UI: $61,900 to $247,000).

Vaccination of boys was not found to be cost-effective, even for additional scenarios with very low vaccine or program administration costs – only when combined vaccine and administration costs were NZ$125 or lower per dose was vaccination of boys cost-effective.

**Conclusions:**

These results suggest that adding boys to the girls-only HPV vaccination program in New Zealand is highly unlikely to be cost-effective. In order for vaccination of males to become cost-effective in New Zealand, vaccine would need to be supplied at very low prices and administration costs would need to be minimised.

## Background

Most developed countries have now implemented vaccination against human papillomavirus (HPV) infection of pre-adolescent girls. This development has been supported by cost-effectiveness analyses in over 40 countries that have almost universally concluded that vaccination of girls is cost-effective [1]. In low resource settings, the GAVI alliance has announced that it will co-finance the HPV vaccine in the poorest countries, with supplier-agreed vaccine prices of about US$5 per dose. In addition to the benefits to women of reduced cervical intra-epithelial neoplasia (CIN) and cervical cancer, HPV vaccination can reduce other cancers and diseases which impact both sexes, including anal and oropharyngeal cancers and genital warts. As such, some public health professionals and researchers profile the ethical debate of the exclusion of boys in a vaccination program from which they could reap additional health benefits. In recent decades, data suggest that incidence rates for HPV-related anal and oropharynx cancer, affecting both sexes, have been increasing in many countries, including the US [2], and Australia [3,4] and markedly in 50–69 year-old males in New Zealand [5]. Vaccinating males as well as females will confer more benefit than vaccinating females only, but the extent of the incremental benefit will critically depend on coverage in females. Vaccination also substantially decreases disease burden related to genital warts, as reported in Australia [6], Sweden [7], and New Zealand [8,9].

Health authorities, including the Advisory Committee for Immunization Practices for the US Centers for Disease Control and Prevention, have recommended the vaccination of boys [10]. While vaccination of boys has been available through private health services in the US, Australia became the first country to offer publicly-funded HPV boys vaccination (quadrivalent vaccine Gardasil^®^) in 2013 [11], although the cost-effectiveness and equity benefit of this program has been criticized [12]. To date, cost-effectiveness analyses of the inclusion of boys into existing girls’ programs have had varying results. HPV-related disease may be reduced in males either through direct benefits to vaccinated males or herd immunity effects to unvaccinated males due to sexual contact with vaccinated females or males. The cost-effectiveness of adding boys to existing vaccination programs depends on a number of model parameters and assumptions, including: the coverage achieved in females, since this will determine the predicted herd immunity (e.g., in dynamic models) for heterosexual males derived from vaccinating girls [13]; the HPV-related burden of disease in males (which is lower than in females [1]); the proportion of HPV-related disease in men-who-have-sex-with-men (MSM; difficult to model for young cohorts and rarely explicitly included in population models) [1]; the cost of the vaccine and administration; the vaccine efficacy and duration of protection; the number of diseases included in analyses, and the associated burden of those diseases in the setting of interest.

When vaccine coverage is high (e.g., over 70%) in females, modeling indicates that non-vaccinated heterosexual males would receive high levels of health benefits via herd immunity [13-15]. For example, in a dynamic Canadian heterosexual population model with a vaccination coverage of 70% for 12-year-old girls and 0% of boys, HPV-16/18 overall population prevalence was reduced by 64% [13]. Adding 70% coverage for boys to this program only decreased this HPV-16/18 prevalence by another 24%. Therefore additionally vaccinating boys, at higher coverage rates, was *less* cost-effective than increasing coverage for girls [13].

For lower vaccination coverage levels (e.g., <50%) one study found that increased coverage for females was more cost-effective than adding boys to a girls-only vaccination program, even if the intensified girls program incurred high costs of US$350 per additional girl vaccinated [16]. A recent review of HPV vaccination cost-effectiveness modeling concluded that increasing female coverage appears to be a better investment, but that vaccination of boys may be cost-effective when female coverage is less than 50% and it costs about US$400-500 per vaccinated girl/boy [1].

Among MSM, herd immunity benefits are obviously lower for girls-only vaccination and depend in part on behavior patterns for non-exclusive MSM in the population. Targeted vaccination of MSM may be cost-effective in some settings (e.g., those with high HIV rates), particularly at younger ages [17]. However, targeted HPV vaccination of a young cohort prior to HPV exposure of MSM is a considerable challenge for vaccination programs to achieve (and we know of no settings where it has been effectively operationalized).

Another important determinant of the cost-effectiveness is vaccine and associated administration costs. Many governments have negotiated vaccine prices with suppliers for large-scale public programs and these costs are often not readily available to the public (although vaccine prices per dose of about US$100 have been used in past studies [1]). Importantly, costs may continue to drop in developed countries and two-dose vaccination (which appear to be nearly as effective as three-dose regimens) may be increasingly introduced [18].

The goal of this research was to examine the cost-effectiveness of adding boys to a girls-only program in New Zealand. This study makes use of national, high-quality health and costing data and includes a number of health outcomes which affect both males and females. We modeled cost-effectiveness for two different coverage rates and also modeled a suite of scenarios for changes in vaccine and administration costs. Such scenarios may facilitate forward-thinking by policy-makers as vaccine prices continue to decline.

## Methods

### Model overview

The study methods followed the Burden of Disease Epidemiology, Equity and Cost-Effectiveness Programme (BODE^3^) Protocol [19]. We adapted a previous Markov model on the cost-utility of girls-only HPV vaccination [20], to estimate quality-adjusted life years (QALYs) gained and net health system costs, for girls-only and girls and boys vaccination. The QALY metric captures both years of life lost from premature death, and loss of quality of life through morbidity. To value this health loss, we used disability weights (DWs), as outlined below. We formally use the term QALY^DW^ in this paper’s Methods and Results, but shorten it to QALY in other sections.

The two main adaptations were adding vaccine and administrative costs per vaccinated boy, and additional reductions in HPV infection due to additionally vaccinating boys.

Health system costs included both direct intervention costs and downstream health system costs incurred/averted. A 3% discount rate applied to costs and quality-adjusted life years (QALYs^DW^) gained. Currency conversions were conducted using the OECD purchasing power parity (PPP) estimates, to eliminate the differences in price levels between countries [21]. In 2011, the PPP values were US$1 = NZ$1.48.

**Figure 1:**
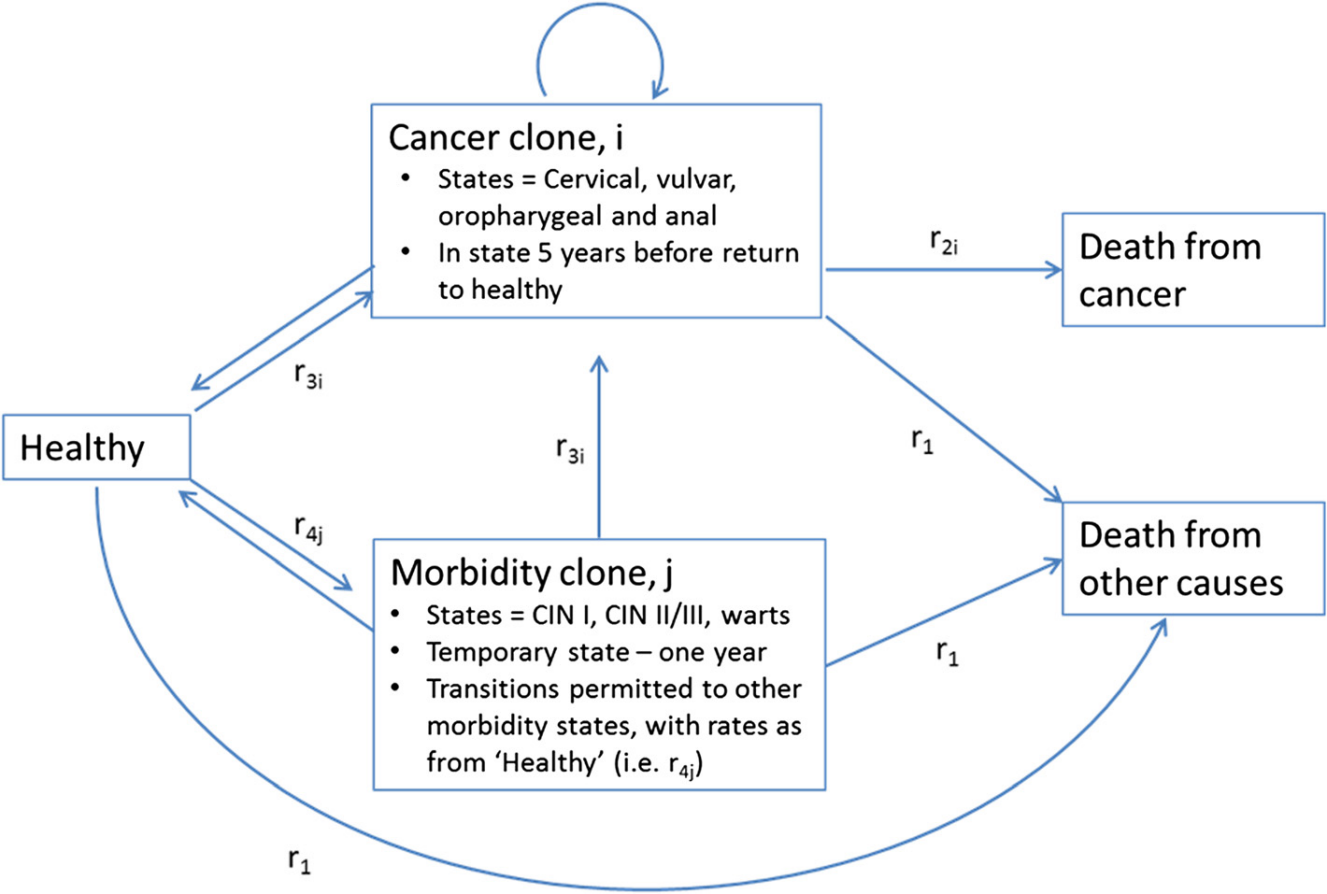
**Stylized Markov model for HPV-related disease states.** r_1_ = rates of all-cause mortality from population lifetables, by sex, age, ethnicity (Māori, non-Māori) and area deprivation (approximate tertiles), and projected to future. Source: [22]. r_2i_ = excess mortality rates of death from cancer i, by sex, age, ethnicity and deprivation, and by time since diagnosis. Source: [23] r_3i_ = incidence rates for cancer i, by sex, age, ethnicity and deprivation. Source: [24] r_4j_ = incidence rates for morbidity states j, by sex and age (and ethnicity for CIN I, CIN II/III and anogenital warts). Source: [20].

HPV vaccination was modeled as preventing CIN I-III, cervical, anal, oropharyngeal and vulval cancers and anogenital warts (Figure 1) using rates of all-cause mortality, excess mortality rates from cancer [22], and incidence rates for cancer [23] and morbidity states [24]. Vaginal and penile cancers were excluded due to their small contribution to the HPV16/18-related cancer burden (<3%). Recurrent respiratory papillomatosis (RRP) was excluded due to sparse data. Cervical cancer screening programs were assumed to continue in New Zealand, but these costs were excluded from our analyses.

### Model input parameters

Input parameters are shown in Table 1 (HPV prevalence reduction and vaccination costs) and Additional file 1: Tables S1 and S2, and briefly described below.

**Table 1:** Intervention parameters: vaccination coverage of 12-year olds, reduction in future HPV and intervention costs

**Intervention**	**Vaccination coverage**	**Beta distribution for vaccination coverage**	**Reduction in HPV infection for central estimate of vaccination coverage only (95% uncertainty interval)**				**Vaccination costs (NZ$ 2011; SD as% of expected valued used for gamma distribution)**
			**Females**		**Males**		
			**HPV6/11**	**HPV16/18**	**HPV6/11**	**HPV16/18**	
**Intervention 1G:** Girls only program as per NZ in 2011	Māori: 56%; assumed SD = 2%	alpha = 344, beta = 271	Māori: 75% (57% to 83%)	Māori: 49% (41% to 59%)	Māori: 75% (55% to 83%)	Māori: 47% (41% to 53%)	$760 (10%)
	Non-Māori: 45%; assumed SD = 2%	alpha = 278, beta =340	Non-Māori: 67% (48% to 76%)	Non-Māori: 41% (33% to 50%)	Non-Māori: 66% (47% to 74%)	Non-Māori: 37% (32% to 43%)	*[($113 vaccine + $141 administrative) × 3]*
**Intervention 2G:** Enhanced uptake as per Australia with school-only delivery (girls only)	73% (no variation by ethnicity or deprivation level); assumed SD = 5%	alpha = 56.8, beta = 21.0	81% (64% to 88%)	63% (53% to 73%)	81% (65% to 88%)	61% (53% to 67%)	$716 (10%) *[($113 vaccine + $126*
							*administrative) × 3]*
**Intervention 1G + B:** Adding boys to 1G	Māori: 56%; assumed SD = 2%	alpha = 8.14, beta = 1.86	Māori: 77% (59% to 85%)	Māori: 67% (53% to 79%)	Māori: 78% (58% to 88%)	Māori: 73% (63% to 83%)	$760 (10%)
	Non-Māori: 45%; assumed SD = 2%	alpha = 8.09, beta = 1.90	Non-Māori: 70% (50% to 79%)	Non-Māori: 58% (45% to 71%)	Non-Māori: 71% (51% to 80%)	Non-Māori: 65% (54% to 75%)	*[($113 vaccine + $141 administrative) × 3]*
**Intervention 2G + B:** Adding boys to 2G	73%; assumed SD = 5%	alpha = 7.84, beta = 2.16	81% (67% to 89%)	78% (65% to 90%)	82% (68% to 89%)	85% (65% to 96%)	$716 (10%)
							*[($113 vaccine + $126 administrative) × 3]*

### Morbidity

Disability weights (DWs) for each disease state were used, with uncertainty, on a scale from 0 (full health) to 1.0 (death) (Additional file 1: Table S1). Expected population morbidity (i.e., due to disease other than HPV-related disease) was allowed for by using the average ethnic and age-specific prevalent years of life lived in disability from the New Zealand Burden of Disease Study [19], limiting the maximum QALYs^DW^ gained with increasing age. Consider a non-Māori male aged 50–54 with oropharyngeal cancer in the remission phase (expected DW = 0.248, although actually modeled with uncertainty), with an expected background morbidity of 0.112 [19]; his expected QALYs^DW^ awarded in that year would be (1–0.248) × (1–0.112) = 0.668.

### Incidence and survival

Cancer incidence rates were those predicted for 2011 with trends projected into the future based on regressions on New Zealand Cancer Registry data [25]. Therefore, when the model was run without intervention effects, the model produced the same disease data as the input dataset. Incidence rates for other diseases and the proportion of disease due to HPV 16/18 or 6/11 were compiled from various sources, including national cancer registration data [24] and screening program information [26]. Australian burden of disease models were used to allocate durations for each cancer in diagnosis and treatment, remission, pre-terminal and terminal states, with attendant DWs sourced from the Global Burden of Disease 2010 [27] with modification to the New Zealand distribution of cancers [19]. Cancer survivors were modeled in the cancer state for five cycles accumulating QALYs with morbidity adjustment via the DWs, then returned to the healthy state.

### Health system cost parameters

Just as QALYs^DW^ were awarded to each individual as they traveled through states, so were health system costs. We used routine, linked administrative health data for the entire New Zealand population with costs attached, as described elsewhere [19]. We assigned health system costs by sex and age to the healthy state (see Additional file 1: Table S2). The added cost for cancer patients at different stages of care (i.e., diagnosis, remission, terminal) were estimated using gamma regression, as per the previous research in the BODE^3^ Program for girls-only vaccination [20]. The added cost for other disease states (CIN I-III and warts) were simple averages. All health system costs were measured in 2011 New Zealand dollars.

### Interventions

#### ***Effectiveness: vaccination coverage and future reduction in HPV prevalence***

The vaccination coverage levels were assumed to be the same for both boys and girls for two interventions: (1G and 1G + B) the current girls’ 3-dose coverage level in 12–13 year olds in New Zealand (45-56%) [28]; and (2G and 2G + B) Australia’s coverage level of 12–13 year old girls (73%), with no catch-up vaccination modeled. We suspect that similar coverage is a reasonable assumption for school-based vaccination programs, provided that sufficient information is communicated to parents and providers about the direct benefits to males of HPV vaccination. We note that a recent review found a preference to vaccinate females over males among both health care providers and parents [29], which could lead to lower coverage for boys than girls. However, the review authors noted that in many studies the direct benefits to males were not communicated and the main reason given for refusal was the lack of perceived direct benefit for males.

The reduction in HPV infections for varying programs of girls-only and girls and boys vaccination was estimated by meta-regressions on outputs from Brisson et al’s (2011) dynamic Canadian model [13], which allowed for changing likelihood of acquiring HPV over time and herd immunity effects. This model used gender and age-specific sexual behavior characteristics (e.g., partner acquisition rates, mixing between age groups) as risk factors for HPV infection. Thus, our Markov macro-simulation models assumed similar sexual behaviors to that in the Canadian model. We considered this generally reasonable on the basis of available comparable data on age of sexual debut and the epidemiology of genital warts. While data on adolescent sexual behavior are sparse, age of sexual debut are similar between Canada [30] and New Zealand [31]. Likewise, incidence of genital warts peak in the same age groups (<25 years old) in both countries [32,33].

Briefly, we fitted regression models to their output for the median, 10^th^ and 90^th^ percentile reductions in HPV prevalence with vaccine coverage as an independent variable for two types of HPV (6/11 and 16/18) for girls-only vaccination, doubled the uncertainty range on the logistic scale to account for ‘structural’ uncertainty when using the results in New Zealand, and then used these median and widened uncertainty intervals to generate Beta distribution parameters to sample in the model (method details described in an Appendix to the parallel study on girls-only vaccination [20]). For the marginal impact of boys, we assumed a correlation of 0.5 between the HPV prevalence impacts of girls-only vaccination and the marginal impact of adding boys, and calibrated Beta distributions to achieve the uncertainty reported in [13] for the marginal impact of adding boys’ vaccination. Examples of future long-term HPV reduction for specified vaccine coverage levels are shown in Table 1. We assumed that the vaccine efficacy was 99% and had a duration of 20 years, that the effect of HPV vaccination was in a ‘steady state’ and that vaccine immunogenicity was the same in both boys and girls.

### Intervention costs

The vaccination costs were calculated per fully vaccinated girl/boy, based on the annual vaccine cost paid by the Ministry of Health (in 2011), resulting in the vaccine cost per dose of NZ$113 (Table 1). The delivery/administration costs of three main interventions were NZ$141 or NZ$126 per dose depending on the method of delivery (i.e., in schools and primary care settings or in schools only). These costing data were based on official Ministry of Health data which include funding to cover program management (a component which is often omitted from ‘administration costs’ of other studies). The vaccination costs were multiplied by three doses, and then assigned for each fully vaccinated member of the cohort (allowing for vaccine coverage); incomplete vaccine courses were not considered. We used a discount rate of 3% and as for health system costs, all intervention costs were measured in 2011 New Zealand dollars.

In scenario analyses, we explored reductions in vaccine cost-per-dose at several levels of reduction down to $1 and lower administrative costs (NZ$19 per dose). We also conducted a scenario analysis which removed the herd immunity cancer reduction benefits for males in the girls-only vaccination program (1G). We compared these results with the benefits of adding boys to the vaccination program (1G + B) and included MSM-attributable warts and cancers in the disease incidence data that populates the model. We also performed a threshold analysis to estimate the maximum cost per delivered vaccine dose (including vaccine and administration costs) which would allow vaccination of boys in addition to girls to be cost-effective compared to girls only vaccination.

### Considering uncertainty and performing cost-effectiveness analysis

Monte Carlo simulation was used, with 2,000 draws from input parameter distributions. Incremental QALYs^DW^, costs and incremental cost-effectiveness ratios (ICERs) were calculated for each draw of Monte Carlo simulation. All modeling and uncertainty analyses were undertaken in TreeAge Pro 2012. ICERs were calculated for each intervention including boys (1G + B and 2G + B), compared to the equivalent intervention for girls-only (1G and 2G) and compared to no vaccination program. In line with contextualizing ICER results with GDP per capita comparisons [34], we made such comparisons for the New Zealand setting. Because there is no universally accepted threshold in New Zealand for describing interventions as being “cost-effective” or not, we relied on the WHO definition and used a nominal GDP per capita of NZ$45,000 in 2011 (US$29,600) as being such a threshold. Using the GDP per capita level is based on the WHO description of interventions below this value as being ‘very cost-effective’ (for the WPROA Region to which New Zealand belongs [34]).

## Results

The modeling results indicated that the estimated intervention costs rose with increasing coverage levels and when adding boys to the girls-only programs (Table 2). QALYs^DW^ gained increased with higher coverage levels. Adding boys to the 2011 girls-only program (assuming the same level of coverage was obtained in boys) produced a similar number of QALYs^DW^ gained (350) as the intensive girls-only program (coverage 73%), but at a greater cost and thus was dominated. The vaccination of boys at the current coverage level for girls would achieve additional health benefits at a cost of NZ$117,500 (95% UI: $57,100 to $215,000; US$61,400) per QALY^DW^ gained compared to the current girls-only program (1G + B compared to 1G). Adding boys to an intensified girls-only program was not cost-effective (2G + B compared to 2G; NZ$247,000 per QALY^DW^ gained, 95% UI: $119,000 to $474,000; US$128,000).

**Table 2:** **Total population level costs, QALYs**^**DW **^**gained and ICERs (95% uncertainty intervals) arising from vaccinating 12-year-olds (boys and girls) in New Zealand in 2011, for the two interventions each compared to no vaccination program and for incremental comparisons**

	**Each intervention compared to no HPV vaccination**				**Incremental comparisons**			
	***Intervention 1G: *** **Replicating the *girls-only *** **NZ program in 2011**	***Intervention 2G: *** **Intensive *girls-only *** **program, school-based**	***Intervention 1G + B: *** **Adding *boys *** **to the *girls *** **NZ program in 2011**	***Intervention 2G + B: *** **Adding *boys *** **to the intensive *girls *** **program, school-based**	***[A] Intervention 2G c.f. Intervention 1G***	***[B] Intervention 1G + B c.f. Intervention 1G***	***[C] Intervention 2G + B c.f. Intervention 1G + B***	***[D] Intervention 2G + B c.f. Intervention 2G***
**Cost of intervention (NZ$; 1,000 s)**	$10,332	$14,955	$21,157	$30,632	$4,624	$10,826	$8,252	$15,676
	($8,537 - $12,383)	($11,877 - $18,491)	($17,482 - $25,360)	($24,325 - $37,873)	($829 - $8,656)	($8,945 - $12,977)	($544 - $16,561)	($12,449 - $19,382)
**Net cost (NZ$; 1,000 s)**	$4,644	$7,326	$13,610	$21,474	$2,683	$8,966	$7,864	$14,147
	($2,269 - $7,045)	($3,873 - $11,126)	($9,223 - $18,370)	($14,932 - $28,858)	($-784 - $6,409)	($6,643 - $11,406)	($296 - $15,921)	($10,588 - $17,975)
**QALYs**^DW^**gained**	267	350	350	413	83	83	63	63
	(162–413)	(218–530)	(222–529)	(266–608)	(48–127)	(47–134)	(19–115)	(33–104)
ICER*	$18,800	$22,300	$41,100	$54,600	$33,500	$118,000	$148,000	$247,000
	($6,500 - $36,700)	($9,500 - $41,100)	($20,700 - $70,000)	($29,900 - $90,400)	($-10,700 - $88,600)	($57,100 - $215,000)	($1,300 - $438,000)	($119,000 - $474,000)

*ICERs rounded to nearest $100 or nearest $1,000 if > $100,000. Discount rate 3%.

**Figure 2:**
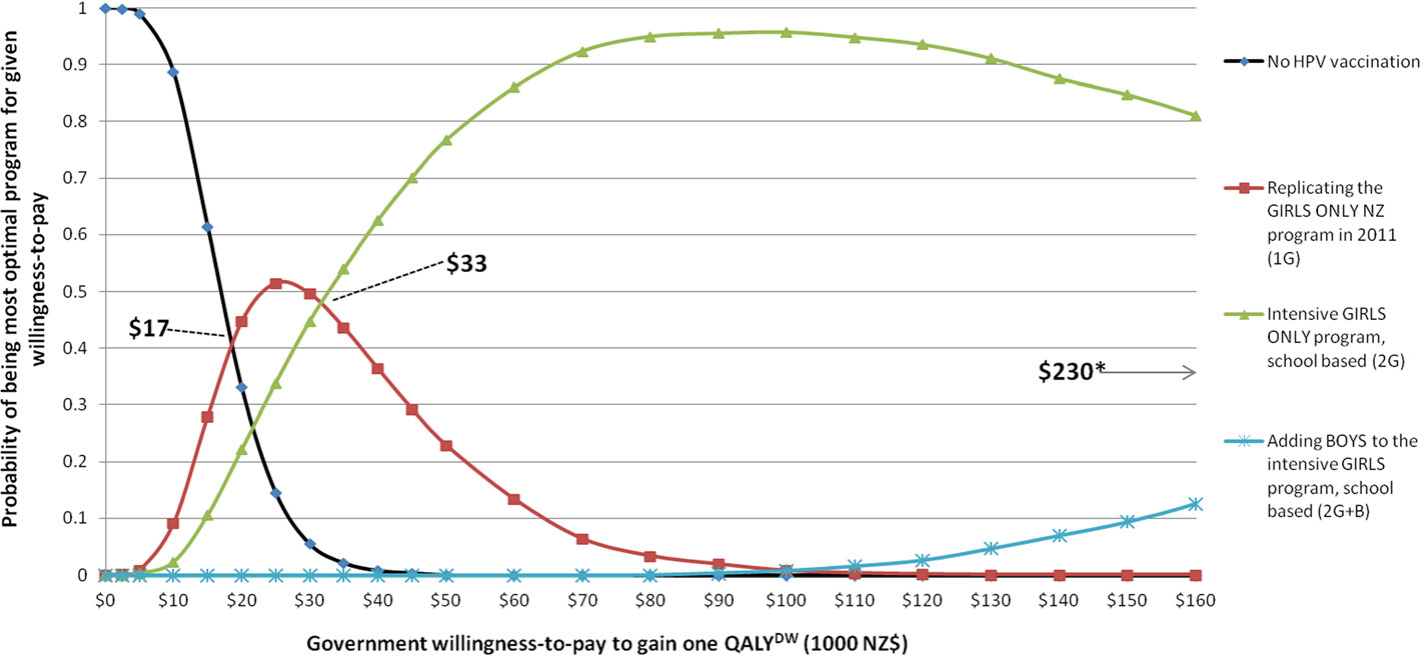
**Multiple cost-effectiveness acceptability curves for three HPV vaccination programs and no HPV vaccination.** *when 2G + B becomes preferred.

**Figure 3:**
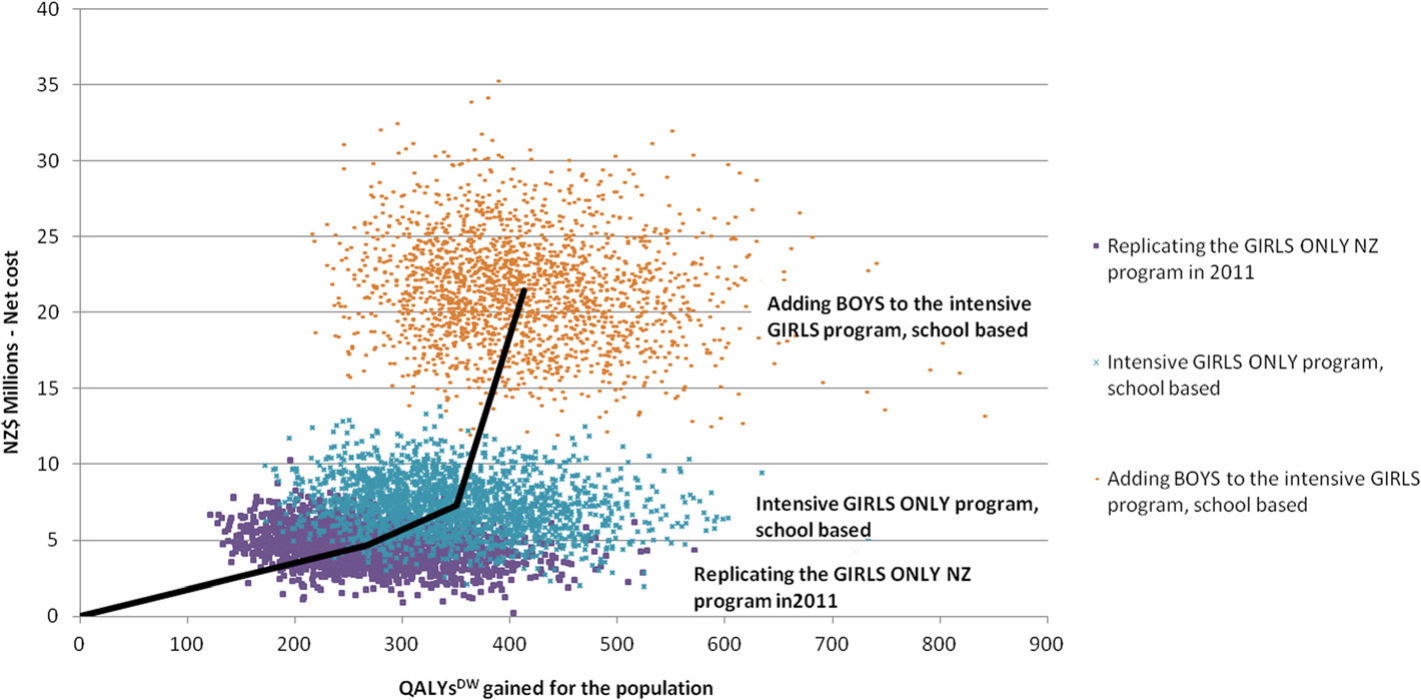
Cost-effectiveness plane for the two girls-only HPV vaccination programs and adding boys to the intensified girls-only program compared to no HPV vaccination (bold black lines join average values).

Females gained more QALYs^DW^ from vaccination than boys, even when the marginal change was from girls-only to boys’ and girls’ vaccination (Table 3). For example, the 2G + B interventions (i.e., both girls and boys at 73% vaccine coverage) compared to 2G still produced more QALYs^DW^ gained for each individual female than male (0.0015 vs 0.006).Figure 2 shows the cost-effectiveness acceptability curves for the three of the four programs (1G + B does not appear as it was dominated as shown in Figure 3) and no HPV vaccination program. Up to a willingness-to-pay of about NZ$17,000, no HPV vaccination is the optimal decision, then girls-only as currently implemented in New Zealand up to NZ$33,000, and from a willingness-to-pay of NZ$33,000 to NZ$230,000 an intensified girls-only program is optimal. Only above a willingness-to-pay of NZ$230,000 would vaccination of boys be optimal.

**Table 3:** **Incremental costs, QALYs**^**DW **^**gained and ICERs per individual 12-year-old over their whole lives (expected value analysis)**

	**Baseline**	**Each intervention compared to no HPV vaccination**				**Incremental comparisons**		
		***Intervention 1G: *** **Replicating the *girls-only *** **NZ program in 2011**	***Intervention 2G: *** **Intensive *girls-only *** **program, school-based**	***Intervention 1G + B: *** **Adding *boys *** **to the *girls *** **NZ program in 2011**	***Intervention 2G + B: *** **Adding *boys *** **to the intensive *girls *** **program, school-based**	***[A] Intervention 2G c.f. Intervention 1G***	***[B] Intervention 1G + B c.f. Intervention1G***	***[D] Intervention 2G + B c.f. Intervention 2G***
***Net cost (NZ$)***								
Total population	$43,807	$81	$129	$235	$372	$47	$154	$244
***QALYs***^***DW ***^ ***gained (all individuals regardless of vaccination status)***								
Total population	26.2830	0.0045	0.0059	0.0059	0.0069	0.0014	0.0014	0.0010
Males	26.3533	0.0025	0.0032	0.0033	0.0038	0.0007	0.0009	0.0006
Females	26.2092	0.0066	0.0087	0.0085	0.0102	0.0021	0.0019	0.0015
ICER*								
Total population		$18,100	$21,900	m	$53,700	$34,000	$111,000	$234,000

*All ICERs rounded to nearest $100 or nearest $1,000 if > $100,000.

**Figure 4:**
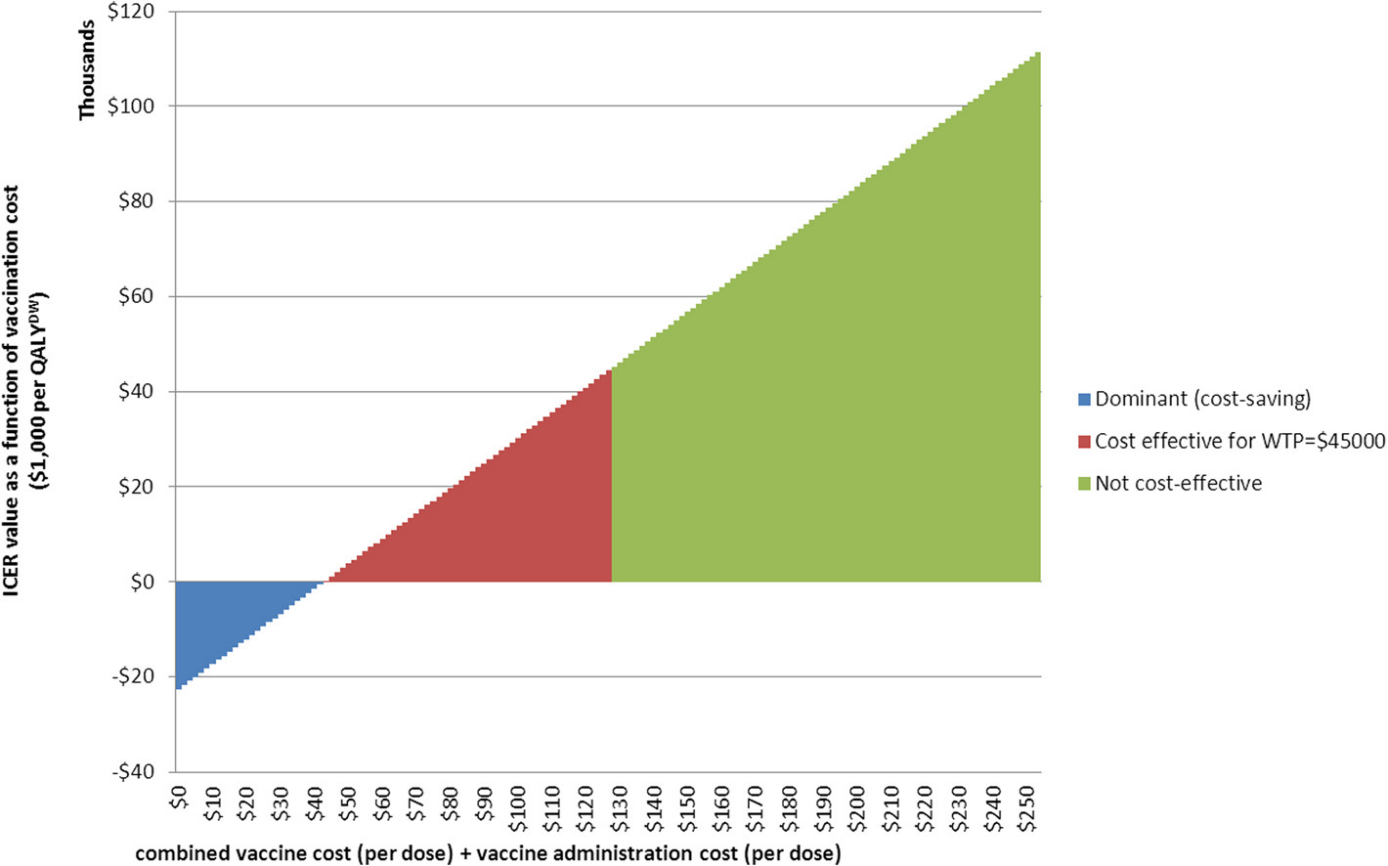
**Cost threshold analysis for the combined vaccine + administration costs per dose for the best model adding boys to the girls-only program.** Incremental cost-effectiveness ratio of adding boys to the current girls-only program (1G + B), compared to the current girls-only program (1G), as a function of cost per dose delivered (including vaccine and administration costs).

Scenario analyses indicate that regardless of either very low vaccine costs or low administrative costs, adding the vaccination of boys was not found to be cost-effective (Table 4). Increasing coverage in girls (2G) was consistently more cost-effective than including boys. Applying a 0% discount rate to costs and QALYs^DW^ still did not lead to the boys programs being cost-effective compared with girls-only programs. However, by combining both very low cost vaccine (NZ$1) with low administration costs, all incremental comparisons including boys compared to girls’ vaccination became dominant. In a threshold analysis, we found that vaccination of boys in addition to girls only became cost-effective (at a willingness-to-pay threshold of $45,000 per QALY^DW^ gained) when the combined administration and vaccine costs were $125 per dose or less (Figure 4), corresponding to an estimated maximum cost per vaccinated individual of $375.

**Table 4:** **Scenario analyses (expected value analysis; average costs and QALYs**^**DW **^**gained per individual 12-year-old over their whole lives**

		***Each intervention compared to no HPV vaccination***				***Incremental comparisons***		
**Interventions**	**Output**	***Intervention 1G: *** **Replicating the *GIRLS-ONLY *** **NZ program in 2011**	***Intervention 2G: *** **Intensive *GIRLS-ONLY *** **program, school-based**	***Intervention 1G + B: *** **Adding *BOYS *** **to the *GIRLS *** **NZ program in 2011**	***Intervention 2G + B: *** **Adding *BOYS *** **to the intensive *GIRLS *** **program, school-based**	***[A] Intervention 2G c.f. Intervention 1G***	***[B] Intervention 1G + B c.f. Intervention1G***	***[D] Intervention 2G + B c.f. Intervention 2G***
From Table 3, expected value only analysis	Net cost (NZ$)	$81	$129	$235	$372	$47	$154	$244
	QALYs^DW^ gained	0.0045	0.0059	0.0059	0.0069	0.0014	0.0014	0.0010
	ICER	$18,100	$21,900	$40,000	$53,700	$34,000	$111,000	$234,000
Vaccine price halved (NZ$56)	Net cost (NZ$)	$41	$68	$154	$248	$27	$112	$180
	QALYs^DW^ gained	0.0045	0.0059	0.0059	0.0069	0.0014	0.0014	0.0010
	ICER	$9,200	$11,600	$26,200	$35,800	$19,000	$81,300	$173,000
Very low vaccine price ($7.46) = GAVI price ~ US$5)	Net cost (NZ$)	$7	$16	$84	$142	$9	$76	$126
	QALYs^DW^ gained	0.0045	0.0059	0.0059	0.0069	0.0014	0.0014	0.0010
	ICER	$1,600	$2,800	$14,300	$20,500	$6,500	$55,300	$121,000
Highly hypothetical vaccine price $NZ 1	Net cost (NZ$)	$3	$10	$75	$129	$7	$72	$119
	QALYs^DW^ gained	0.0045	0.0059	0.0059	0.0069	0.0014	0.0014	0.0010
	ICER	$700	$1,700	$12,800	$18,600	$4,900	$52,200	$115,000
Plausibly lower vaccine administration costs (NZ$19)	Net cost (NZ$)	**$-4**	$14	$61	$138	$18	$65	$124
	QALYs^DW^ gained	0.0045	0.0059	0.0059	0.0069	0.0014	0.0014	0.0010
	ICER	*Dominant*	$2,400	$10,400	$20,000	$12,800	$47,000	$119,000
Hypothetical vaccine price NZ$1 + lower vaccine administration costs (NZ$19)	Net cost (NZ$)	**$-80**	**$-103**	**$-99**	**$-110**	**$-22**	**$-18**	**$-7**
	QALYs^DW^ gained	0.0046	0.0059	0.0058	0.0069	0.0013	0.0012	0.0010
	ICER	*Dominant*	*Dominant*	*Dominant*	*Dominant*	*Dominant*	*Dominant*	*Dominant*
GAVI vaccine price + lower vaccine administration costs (NZ$19)	Net cost (NZ$)	**$-77**	**$-99**	**$-90**	**$-94**	**$-22**	**$-12**	$5
	QALYs^DW^ gained	0.0045	0.0059	0.0059	0.0069	0.0014	0.0014	0.0010
	ICER	*Dominant*	*Dominant*	*Dominant*	*Dominant*	*Dominant*	*Dominant*	$5,000
Discount rate 0%	Net cost (NZ$)	**$-28**	**$-23**	$80	$183	$5	$108	$205
	QALYs^DW^ gained	0.0135	0.0186	0.0190	0.0233	0.0051	0.0055	0.0047
	ICER	*Dominant*	*Dominant*	$4,200	$7,900	$1,000	$19,400	$43,900
Cost and QALYs^DW^ discount rate 6% (double baseline)	Net cost (NZ$)	$123	$186	$293	$443	$63	$170	$257
	QALYs^DW^ gained	0.0025	0.0031	0.0030	0.0035	0.0006	0.0006	0.0004
	ICER	$50,100	$59,900	$96,500	$127,000	$97,000	$294,000	$695,000
Excluding unrelated health system costs †	Net cost (NZ$)	$73	$117	$222	$356	$44	$150	$240
	QALYs^DW^ gained	0.0045	0.0059	0.0059	0.0069	0.0014	0.0014	0.0010
	ICER	$16,200	$19,800	$37,900	$51,500	$31,500	$109,000	$231,000
Excluding disease DWs (i.e., no morbidity impacts of HPV-related disease) ^	Net cost (NZ$)	$81	$129	$235	$372	$47	$154	$244
	QALYs^DW^ gained	0.0011	0.0016	0.0017	0.0022	0.0006	0.0007	0.0006
	ICER	$76,500	$79,800	$137,000	$166,000	$86,200	$233,000	$383,000
Excluding both background morbidity and disease DWs (i.e., life years gained analysis, ignoring morbidity)	Net cost (NZ$)	$81	$129	$235	$372	$47	$154	$244
	QALYs^DW^ gained	0.0015	0.0023	0.0024	0.0031	0.0008	0.0009	0.0009
	ICER	$53,100	$56,300	$96,700	$119,000	$62,700	$171,000	$284,000
Excluding herd immunity benefits related to anal and oropharyngeal cancers for males when only females vaccinated: 1G (i.e., considering underestimation of benefits to MSM in 1G + B)	Net cost (NZ$)						$144	
	QALYs^DW^ gained						0.0018	
	ICER						$80,000	

† That is ignoring the health costs from diseases other than those specifically modeled, which increase net costs as living longer is associated with costs from (other) future disease and disability.

^ That is the DWs for cancers, CIN and anogenital warts states are all set to zero – but the background morbidity is retained. The health gain realized from HPV vaccination is therefore only from preventing premature death from cancer. NOTE: all items in bolded font are cost-saving.

We undertook sensitivity analyses to explore which input parameter uncertainty was driving most of the uncertainty in outputs (i.e., net cost, QALYs^DW^ gained and the ICER) (see Additional file 1: Figure S1). In Table 4, we explored possible underestimation of comparative advantage for MSM in the boys’ and girls’ vaccination program (1G + B) compared to girls only (1G). To do this, we took an extreme case and removed all of the herd immunity cancer reduction benefits (i.e., anal and oropharyngeal) for males in the 1G program and compared this to the benefits of our ‘best’ 1G + B model (i.e., with anal and oropharyngeal male cancer benefits reintroduced. In this analysis the estimated ICER was $80,000 per QALY^DW^ gained, which is still far above a willingness-to-pay threshold of $45,000. Additionally, a Tornado Plot for the ICER for 1G + B compared to 1G is shown in Additional file 1: Figure S1. Uncertainty about the parameter for marginal HPV reduction from vaccinating boys in addition to girls was the biggest driver of uncertainty, but even using the 97.5^th^ percentile value of these marginal proportionate reductions in HPV (i.e., assuming the greatest incremental benefit for adding boys vaccination), the ICER was still greater than $75,000 per QALY^DW^ gained (holding all other parameters at their expected value). In a cost threshold analysis for the most favourable and extreme scenario for boys’ vaccination (excluding herd immunity benefits related to anal and oropharyngeal cancers for males when only females vaccinated), we found that the combined cost of the vaccine and administration would need to be $167 per dose or lower (i.e., 34% less than best model expected price of $254 and 34% higher than best model’s cost-effectiveness threshold price of $125 ) for vaccination to be considered cost-effective at a willingness-to-pay threshold of NZ$45,000/QALY^DW^ (see Additional file 1: Figure S2).

## Discussion

### Discussion of main findings and interpretation

Our finding that HPV vaccination of boys was not cost-effective when vaccination coverage in a girls-only program was high (>50%) was consistent with other modeling on the incremental cost-effectiveness of adding boys to girls-only vaccination programs [1,35]. Specifically, we found that providing vaccination of boys at the current coverage level for girls would cost US$61,400 per QALY gained (NZ$118,000, 95% UI: $57,100 to $215,000) compared to the current girls-only program. For comparison to other international studies, it is useful to use US dollars. In the base case, we assumed the current New Zealand vaccine cost of US$59 (NZ$113) per dose and administrative costs of US$73 (NZ$141) per dose. By comparison, US models (which do not generally assume vaccine is delivered as a publicly-funded health service) assumed vaccine costs (in 2011 USD) of US$101-135 per dose [35,36] and US$61 administration costs per dose [35]. Those same studies, when comparing the addition of boys to girls-only vaccination programs, and including all HPV-related diseases and similar vaccine efficacy and duration to our study, were found to be more cost-effective than our study, ranging from US$26,000 [36] to $41,000 [35] per QALY for coverages under 50% and $62,000/QALY for coverages up to 70% [37]. While direct comparisons between cost-effectiveness analyses are difficult, the observed differences in cost-effectiveness are most likely due to varying disutility assigned to genital warts and CIN states (less in our model than in the US studies, see Additional file 1: Table S1; and uncertainty analyses in our parallel girls-only vaccination paper demonstrated that disutility assigned to these states was a major driver of uncertainty in the final ICER [20]). Also, the inclusion of future health system costs in our model will slightly lessen cost-effectiveness (scenario analyses about excluding unrelated health system costs show little impact, see Table 4) and our use of a cohort (rather than a population-wide) model may have influence. Our study also found that vaccination of boys only became cost-effective under the conditions of both an extremely low vaccine price (NZ$1 per dose) and with very low administration costs (NZ$19 per dose), up to a maximum total cost of NZ$125 per dose delivered. However, even at a lower cost per dose delivered, because a more intensive female-only program is equally effective and less costly than adding boys to the current program, increasing coverage in girls would generally remain a preferable strategy if this could be achieved.

### Study strengths and limitations

A strength of this study is its potential to fill a gap in the knowledge base for New Zealand policy makers who may be considering what next after the relatively successful implementation of a girls-only HPV program. More broadly it adds to the relatively small number of studies that have addressed the cost-effectiveness of vaccinating boys in a publicly funded system.

Nevertheless, a limitation is the lack of local data to build a dynamic HPV infection model and thus, our reliance on HPV reduction results from a Canadian dynamic model. Thus we implicitly assumed that sexual behavior in New Zealand and Canada are broadly similar for reasons outlined in the Methods section. This should not be overly problematic if vaccine coverage rates are not correlated with level of sexual behavior or factors which influence choice of sexual partner [38]. Furthermore, refined and improved HPV modeling specific to New Zealand would probably not alter our conclusion that vaccination of boys is not cost-effective using a NZ$45,000 per QALY threshold (see Additional file 1: Figure S1). Rather, a reduction in vaccine cost – perhaps plausible in the future – is more likely to influence whether vaccination of boys is cost-effective in New Zealand (see Table 4 and Additional file 1: Figure S1). That is, uncertainty in applying Brisson et al. outputs (based on their model assumptions, Canadian sexual contact patterns, etc.) to the New Zealand situation – whilst important – does not appear to be enough to alter the general conclusion that vaccinating boys in New Zealand is not cost-effective (in the absence of large vaccination price reductions). Another related issue was our use of a ‘steady state’ of HPV reduction in our population model. Our model estimates likely health impacts, costs and cost-effectiveness for cohorts of 12-year-olds one to three decades into the future after the first cohort is vaccinated. This is a robust baseline model for the following reasons. First even for the first cohort vaccinated, they will still receive the benefits of direct immunity (i.e., the majority of the benefit from HPV 16/18 reduction, as herd effects are smaller for these HPV types, and also much of benefit from HPV 6/11 reduction, which has greater herd immunity [13]). The first cohort vaccinated will also receive the benefit of herd immunity from vaccination of the cohort’s immediate age contemporaries and subsequent vaccination of younger cohorts and additionally some herd immunity from older cohorts included in the catch-up program. Girls up to nine years older than the modeled cohort (i.e., born in 1990 or later) were offered vaccination, with achieved three-dose coverage in the range 37-54% [39]. Last, there is a ‘lead time’ benefit. For example, those vaccinated at age 12 years likely have a modal age of coitarche of 15–18 years [30], and a period of maximum exposure to HPV, due to multiple sexual partners, from age 15 to 30 years. This cohort will start to benefit from herd immunity as previous modeling studies e.g., [15] and now observed data from several countries [40] suggest that HPV prevalence is likely to drop rapidly after the introduction of HPV vaccination.

We did not include any costs associated with starting-up a boys’ vaccination program, as these would likely be comparatively small when considered over the longer term and because including boys is extending the existing girls-only program, rather than a new program. (Scenario analyses available from the authors on request confirm this). We also did not model different coverage rates for boys and girls, given we expected minimal differences can be achieved, particularly with school-based vaccination programs. We did not include the health benefits of cross-protection of the quadrivalent vaccine against other HPV strains which cause disease; such analyses would likely have only a minor positive impact on QALYs gained and therefore only modestly affect cost-effectiveness [41]. Our modeling does not separately model MSM, although we did conduct a scenario analysis which removed the herd immunity cancer reduction benefits for males in the girls-only vaccination program (1G). We compared these results with the benefits of adding boys to the vaccination program (1G + B) and included MSM-attributable warts and cancers in the disease incidence data that populates the model. Should a targeted program be able to successfully deliver vaccination to MSM at a young age (e.g., in primary care settings), it seems highly likely that this would be cost-effective [17].

Our costing data for vaccination delivery were based on official Ministry of Health data which include funding to cover program management (a component which does not seem to typically be included in the ‘administration costs’ of other studies). We recognize that this cost and relatively high vaccine costs do not account for future plausible developments (e.g., in changes in delivery (see below) or declines in vaccine prices). The latter is particularly relevant to New Zealand, which has a single government-owned agency (Pharmac) that purchases pharmaceuticals for the whole country and has recently taken over national-level vaccine purchases (from the Ministry of Health). This development could result in substantial reductions in vaccine prices in future purchase arrangements.

### Research implications

The cost-effectiveness results were sensitive to vaccine and administration costs, suggesting an analysis of a vaccination program with only two required doses would be useful in future research. However, if the efficacy of two doses were similar to that of three doses, vaccination of boys would still not be cost-effective based on our findings from our ‘best’ model (i.e., that with current prices), unless the total cost per vaccinated individual was less than NZ$375 (based on our threshold analysis). For example, for incremental comparison B in Table 2 (1G + B c.f.1G) the cost of the intervention (first row) would decrease by a third or $3610, and the net incremental cost would therefore reduce by 40% from $8,996 to $5390. Hence the ICER would also reduce by 40% to about $70,400 per QALY gained.

If new evidence of lower vaccine bulk prices tendered by developed countries becomes available, future modeling may be worthwhile as it is likely to suggest improvements in cost-effectiveness (lower ICERs). Nevertheless, from our analyses, the vaccine price would have to be substantially less than the NZ$113 per dose assumed here to include boys in any vaccination program, and the overall costs of delivering a dose would have to be less than NZ$125, or less than an overall cost per vaccinated individual of NZ$375. Countries with other school-based vaccination programs (e.g., the DTP booster given to 11-year-olds as per the New Zealand schedule) could also explore the administrative cost savings by providing dual vaccination at this one service. Such combinations of ‘piggy-backing’ of administration cost reductions along with reductions in vaccine price appear, in our modeling, to be the major way that vaccination of boys could start to become cost-effective in the current New Zealand setting.

### Possible policy implications

Given the findings of this modeling, policy makers in settings like New Zealand (with relatively low HPV coverage for girls) should probably focus more on improving HPV vaccination for girls than adding HPV vaccination for boys. Indeed, they should probably set decision review dates around HPV vaccination for boys out into the medium term or at least until vaccine prices drop substantially.

If policy makers are under public pressure to provide vaccination for boys then they should ideally focus on ways to reduce the overall cost per vaccinated individual, as this will increase the cost-effectiveness of adding boys to the program, for example via research around cost-reduction measures (e.g., how to reduce delivery costs and also how to obtain lower cost vaccine as detailed in the section above). They could also reassure the public that the initial most logical goal of achieving higher HPV vaccination coverage for girls will actually produce substantial spillover benefits via herd immunity for boys.

## Conclusions

These modeling results suggest that in countries like New Zealand, the health sector would get the best value-for-money by further improving HPV vaccination coverage for girls, rather than adding in the vaccination of boys. Nevertheless, if very low vaccine *and* program administration costs are achieved in the future, vaccination of school boys may become cost-effective.

## Competing interests

KC is co-PI of a new trial of primary HPV screening in Australia (‘Compass’) which has received a funding contribution from Roche Molecular Systems and Ventana Inc. USA. She has sat on Expert Advisory Boards for Merck Inc USA but has accepted no honoraria or expenses for this work.

## Authors’ contributions

NW and TB conceived of the study. GK and AP developed models and conducted simulations. AP led the drafting of the manuscript. All authors provided feedback on the drafts and expert advice. All authors read and approved the final manuscript.

## Pre-publication history

The pre-publication history for this paper can be accessed here:

http://www.biomedcentral.com/1471-2334/14/351/prepub

## Supplementary Material

Additional file 1: Table S1 Input parameters to the modeling: selected base case parameters. **Table S2.** Health system costs for different states of disease in the Markov model. **Figure S1.** Tornado plot showing the impact on the ICER comparing boys and girls vaccination with just girls at about 50% vaccine coverage (1G + B compared to 1G) from using the 2.5^th^ and 97.5^th^ percentile value of each input parameter (from its uncertainty distribution) whilst holding all other input parameters at their expected value, for one population stratum (Māori population, most deprived tertile). **Figure S2.** Cost threshold analysis for combined vaccine + administration costs per dose, for the most favourable and extreme scenario for boys’ vaccination (excluding herd immunity benefits related to anal and oropharyngeal cancers for males when only females vaccinated). Incremental cost-effectiveness ratio of adding boys to the current girls-only program (1G + B), compared to the current girls-only program (1G), as a function of cost per dose delivered (including vaccine and administration costs). Click here for file
