# Supplementary material for: Is expanding HPV vaccination programs to include school-aged boys likely to be value-for-money: a cost-utility analysis in a country with an existing school-girl program
Source: BMC Infect Dis. 2014 Jun 26;14:351. doi: 10.1186/1471-2334-14-351 (PMC4082618; doi:10.1186/1471-2334-14-351)

Supplementary Table S1: Input parameters to the modelling: selected base case parameters

| **Variable** | **Comment and best estimate** | | | **Variation/uncertainty range** | |
| --- | --- | --- | --- | --- | --- |
| ***Disease incidence rates*** |  | | |  | |
| Cervical (females only), and oropharyngeal cancers | By age, ethnicity and deprivation. Projected to 2026, then constant. Source: [[21](#_ENREF_21)] | | | Nil (but scenario analyses about future reductions) | |
| Vulvar cancers (females), anal cancers | By age (data too sparse by ethnicity and deprivation). No projected change. Source: Analyses of NZCR data 2005-2009. | | | Nil | |
| Cervical intraepithelial neoplasia (CIN) I,II,III incidence (females only) | By age and ethnicity. No projected change. Source: [[22](#_ENREF_22)] and other cervical screening program data ([www.nsu.govt.nz](http://www.nsu.govt.nz)). | | | Nil | |
| Anogenital warts | By sex, age and ethnicity. No projected change from the 2007 incidence rate. Source: Multiple. | | | Nil | |
| ***Proportion of disease due to HPV in 2011*** |  | | |  | |
| Cervical cancer (16/18) (females) | A meta-analysis of Australian studies found a prevalence of either HPV 16 or 18 of 77.7% [35]. (An international combined analysis suggest just less than 70% [36].) The SE was 3.8%, which we inflated by 1.2 for uncertainty in application of Australian data to NZ. | | | Beta distribution, alpha=62.23, beta=17.86, generating mean 0.777 and SD=0.046. | |
| Oropharyngeal cancer (16/18) | Smith et al (2011) provide mostly international estimates for ICD codes C01-06, and C09-10 [37]. Applying their central, estimates to NZ 2007-09 cancer registry data, 19.8% of NZ oropharyngeal cancer (ICD C01-14) is estimated as being due to HPV16/18. Using just their minimum/maximum estimates, the range is 2.9% to 28.3%. However, this is likely to substantially overstate uncertainty as it seems unlikely that all minimum (maximum) estimates would apply simultaneously to all sub-cancers. We assumed SD=4% using this data, but inflated it by 1.5 for uncertainty in application to NZ. | | | Beta distribution, alpha=8.54, beta=34.57, generating mean=0.198 and SD=0.06. | |
| Anal cancer (16/18) | 72% (minimum 60%, maximum 83%). Source:[37] | | | Beta distribution: alpha=39.6, beta=15.4, generating mean=0.72 and SD=0.06. | |
| Vulvar cancer (16/18; females) | 32% (no uncertainty provided; assumed SD is about 6%, consistent with other cancers). Source:[38] | | | Beta distribution: alpha=19.0, beta=40.4, generating mean=0.32 and SD=0.06. | |
| CIN (16/18; females) | CIN I: 24.3% (95% CI 23.6-25.0). International data, set SD=1%.  CIN II/III: 44.6% (95% CI 39.3-49.9). Australian data – inflate SE by 1.2.  Sources:[39-41]; <http://apps.who.int/hpvcentre/statistics/dynamic/ico/DataQueryResult.cfm>, accessed 6 Sept 2012) | | | CIN I: Beta distribution: alpha=447, beta=1392, generating mean=0.243 and SD=0.01.  CIN II/III: Beta distribution: alpha=42.2, beta=131.5, generating mean=0.446 and SD=0.032. | |
| Anogenital warts (6/11) | 99% (this value was from a large RCT). | | | Beta: alpha = 99, beta = 1, generating mean=0.99 and SD=0.01. | |
| ***Mortality rates*** |  | | |  | |
| Background mortality rates | From projected life-tables by age, ethnicity and deprivation. Source:[42] | | | Nil | |
| Cervical and oropharyngeal cancer excess mortality rates (EMR) | By age, ethnicity and deprivation, and time since diagnosis. Projected to 2026, then constant. Assume HPV 16/18 attributed cancer have same EMR as all cancer. Assume statistical cure at 5 years post diagnosis, equating to five year tunnel states in Markov model. Source: Table 30 [34] | | | Nil | |
| Anal and vulvar cancer EMRs | Assumptions as for cervical and oropharyngeal cancers above. However, rectal cancer EMRs scaled to give anal cancer EMRs, and cervical cancer EMRs scaled to give vulvar cancer EMRs, as follows: the 5yr RSR for anal cancers in England and Wales was 50.5% c.f. 43.1% for rectal cancers [43], which is equivalent to scaling NZ (Source: Table 30 [34]) rectal EMRs by 0.81; the 5yr RSR for other female genital cancers (1999-2003; 71% were vulvar) in five Scandinavian countries was 58.1% c.f. 65.7% for cervical cancer [44], which is equivalent to scaling the NZ cervical cancer EMRs by 0.77 to give vulvar cancer EMRs. (Workings available from authors on request.) | | | Nil | |
| ***Cancer disability weights and duration*** ^$^ | *Cervical* | *Oropharyngeal* | *Vulvar, anal* | |  |
| Diagnosis and treatment (DT) | 0.288 (0.194;0.404) (3 months) | 0.375 (0.264;0.502) (3 months) | 0.295 (0.200;0.412) (4 months) | |  |
| Pre-terminal (PT) | 0.487 (0.332;0.645) (5 months) | 0.584 (0.424;0.729) (8 months) | 0.487 (0.332;0.645) (8 months) | |  |
| Terminal (T) | 0.495 (0.334;0.656) (1 month) | 0.594 (0.429;0.740) (1 month) | 0.495 (0.334;0.656) (1 month) | |  |
| Remission (R) | 0.134 (0.085;0.306) (residual) | 0.248 (0.164;0.356) (residual) | 0.161 (0.103;0.243) (residual) | |  |
| ***Non-cancer disability weights and utility values (duration)*** |  | | |  | |
| CIN I | Assume plausible range DW of 0 to 0.045. Midpoint 0.0225. Assume SD=0.012. | | | Beta distribution: alpha=3.4, beta=148, generating mean=0.022 and SD=0.012. | |
| CIN II/III | Assume plausible range DW of 0 to 0.065. Midpoint 0.0325. Assume SD=0.016. | | | Beta distribution: alpha=4.0, beta=118, generating mean=0.033 and SD=0.016. | |
| Anogenital warts | DW 0.03 with SD=0.01. | | | Beta distribution: alpha=8.7, beta=281, generating mean=0.03 and SD=0.01. | |

^$^ Disability weights (DW) for all cancers combined taken from the Global Burden of Disease Study 2010 [[23](#_ENREF_23)], then calibrated by cancer [[19](#_ENREF_19)].

NZCR = New Zealand Cancer Registry

Supplementary Table S2: Health system costs for different states of disease in the Markov model

|  | **Females** |  |  | **Males** |  |  |
| --- | --- | --- | --- | --- | --- | --- |
|  | **20 yr** | **40 yr** | **60 yr** | **20 yr** | **40 yr** | **60 yr** |
| ***Population/citizen health system costs per month (no uncertainty modeled)*** |  |  |  |  |  |  |
| Healthy (i.e. average citizen cost by sex and age; per month cost) | $69 | $103 | $234 | $62 | $92 | $208 |
| Average citizen in last six months of life (from cause of death other than cancers in the model) | $1206 | $1226 | $1658 | $996 | $1013 | $1369 |
| ***Cancer excess costs per month (additional cost over and above healthy; no uncertainty modeled) †*** |  |  |  |  |  |  |
| Cervical – diagnosis and treatment | $6990 | $7284 | $5793 | - | - | - |
| Cervical – remission | $1936 | $1950 | $1551 | - | - | - |
| Cervical – pre-terminal | $4075 | $4247 | $3377 | - | - | - |
| Cervical – terminal | $13,539 | $14,108 | $11,221 | - | - | - |
| Oropharyngeal – diagnosis and treatment | $6692 | $6865 | $7102 | $6692 | $6865 | $7102 |
| Oropharyngeal – remission | $1763 | $1793 | $1855 | $1763 | $1793 | $1855 |
| Oropharyngeal – pre-terminal | $4492 | $4569 | $4726 | $4492 | $4569 | $4726 |
| Oropharyngeal – terminal | $13,239 | $13,466 | $13,930 | $13,239 | $13,466 | $13,930 |
| Anal – diagnosis and treatment | $5594 | $5594 | $3987 | $5594 | $5594 | $3987 |
| Anal – remission | $6942 | $6942 | $1440 | $6942 | $6942 | $1440 |
| Anal – pre-terminal | $8245 | $8245 | $4010 | $8245 | $8245 | $4010 |
| Anal – terminal | $12,505 | $12,505 | $8746 | $12,505 | $12,505 | $8746 |
| ***Other disease state costs per year (additional cost over and above healthy; uncertainty distribution in parentheses) ‡*** |  |  |  |  |  |  |
| CIN I | $779 (Gamma, SD $78) | | |  | | |
| CIN II/III | $1431 (Gamma, SD $145) | | |  | | |
| Anogenital warts | $395 (Gamma, SD $20) | | | $257 (Gamma, SD $13) | | |

Costs shown for six sex by age combinations, but regression based estimates used for all ages for population and cancer excess costs)

†Data was too sparse for separate calculations for anal and vulval cancer. Accordingly, costs for colorectal and cervical, respectively, were used. Due to cancer occurring many years after vaccination at age 12, discounting will mean the model is relatively insensitive to these assumptions.

‡ No variation by age modeled.

Supplementary Figure S1: Tornado plot showing the impact on the ICER comparing boys and girls vaccination with just girls at about 50% vaccine coverage (1G+B compared to 1G) from using the 2.5^th^ and 97.5^th^ percentile value of each input parameter (from its uncertainty distribution) whilst holding all other input parameters at their expected value, for one population stratum (Māori population, most deprived tertile)


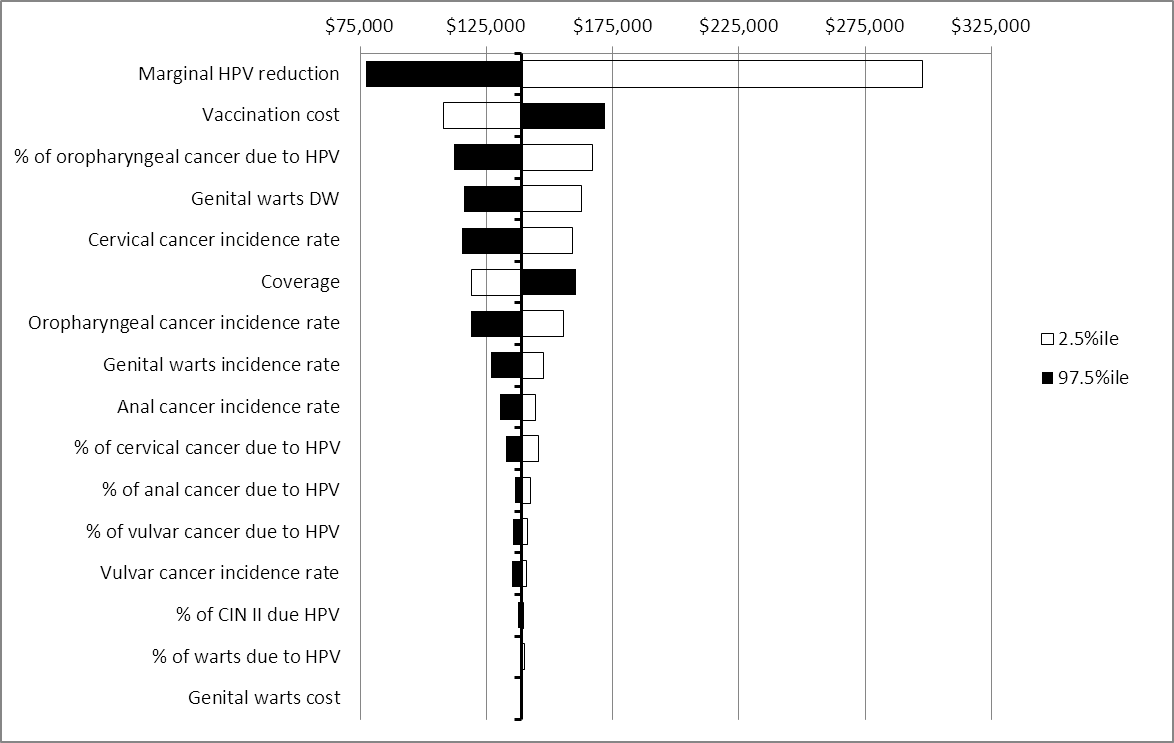


Note: For the Marginal HPV reduction parameter, the 97.5^th^ percentile values for percentage HPV16/18 reduction among boys was 55%, which given that 1G already achieved 45% equates to 55% × 55% = 30% *absolute* increased seroprevalence reduction on top of 1G. For the Marginal HPV reduction parameter, the 97.5^th^ percentile values for percentage HPV 6/11 reduction among boys was 73%, which given that 1G already achieved 70% equates to 30% × 73% = 22% *absolute* increased seroprevalence reduction on top of 1G . These corresponding percentages (absolute marginal gains in parentheses) were an additional 47% (26%) reduction in HPV 16/18 among girls and 73% (22%) for HPV 11/16 among girls. The 2.5th percentile values for absolute HPV seroprevalence reduction were 18%, 1%, 9% and 1% for HPV16/18 and 6/11 among boys, and HPV 16/18 and 6/11 among girls.

Vaccination cost includes both vaccine and administration costs.

Supplementary Figure S2: Cost threshold analysis for combined vaccine + administration costs per dose, for the most favourable and extreme scenario for boys’ vaccination (excluding herd immunity benefits related to anal and oropharyngeal cancers for males when only females vaccinated)

Incremental cost-effectiveness ratio of adding boys to the current girls-only program (1G+B), compared to the current girls-only program (1G), as a function of cost per dose delivered (including vaccine and administration costs)


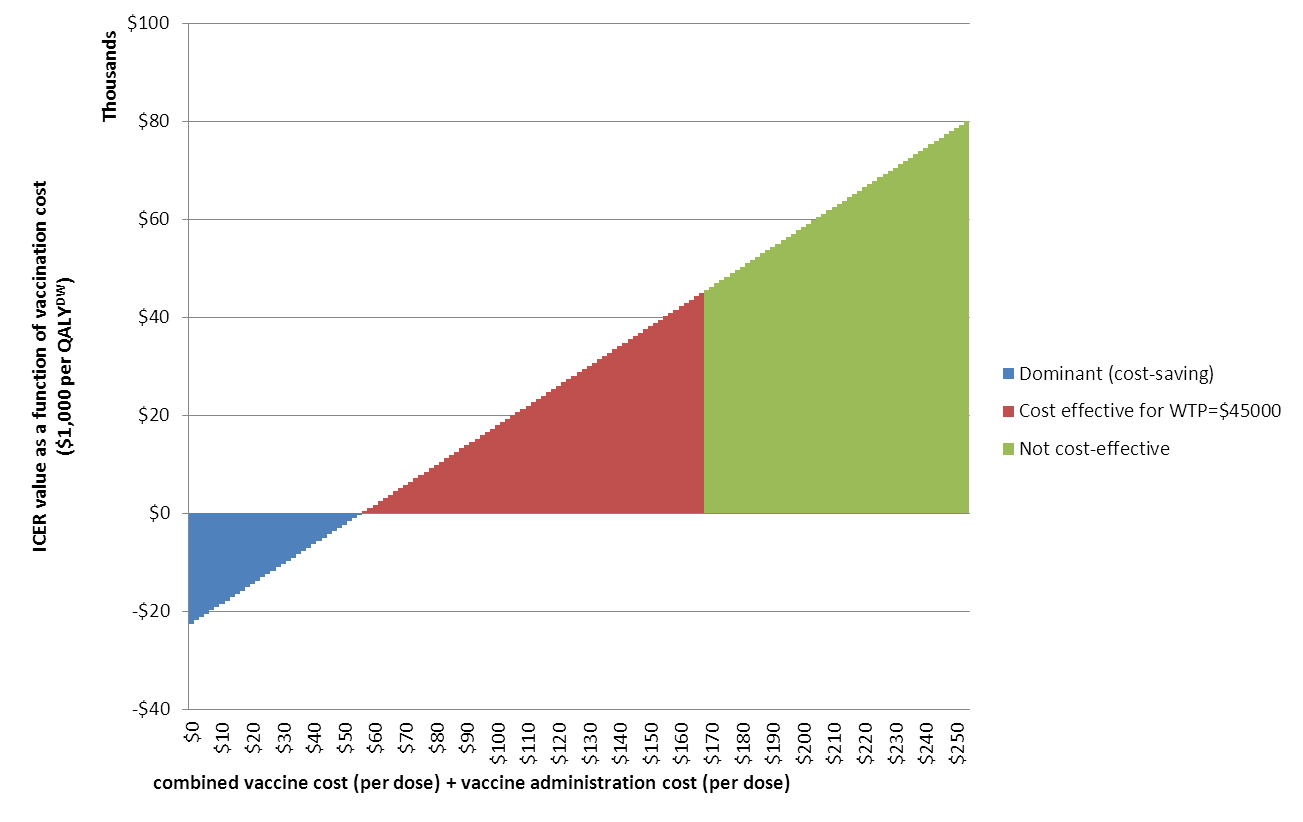

Supplement: Additional file 1: Table S1 — Input parameters to the modeling: selected base case parameters. Table S2. Health system costs for different states of disease in the Markov model. Figure S1. Tornado plot showing the impact on the ICER comparing boys and girls vaccination with just girls at about 50% vaccine coverage (1G + B compared to 1G) from using the 2.5th and 97.5th percentile value of each input parameter (from its uncertainty distribution) whilst holding all other input parameters at their expected value, for one population stratum (Māori population, most deprived tertile). Figure S2. Cost threshold analysis for combined vaccine + administration costs per dose, for the most favourable and extreme scenario for boys’ vaccination (excluding herd immunity benefits related to anal and oropharyngeal cancers for males when only females vaccinated). Incremental cost-effectiveness ratio of adding boys to the current girls-only program (1G + B), compared to the current girls-only program (1G), as a function of cost per dose delivered (including vaccine and administration costs). [file 1471-2334-14-351-S1.docx]
